# State Medical Society

**Published:** 1848-04

**Authors:** 


STATE MEDICAL SOCIETY.

The proposition to form a State Medical Society for Pennsylvania, originally suggested, we believe, by the Chester County Medical Society, has been responded to in a number of the counties. The Lancaster County Medical Society has suggested that the convention for that object shall meet in the city of Lancaster, on the second Tuesday of the present month, (11th inst.,) and both time and place seem to be generally approved. Beside the delegates appointed by the Philadelphia Medical Society, published in our last, we have learned of the following:

*University of Pennsylvania*—Professors Jackson and Gibson.

*Jefferson Medical College*—Professors Mitchell and Mütter.

*College of Physicians*—Drs. Jackson, Hays, Carson, La Roche, Griscom, Rodman, J. F. Meigs, Godon, Littell and F. G. Smith.

*Northern Medical Association*—Drs. Stewart, Maburry, Rhein and Hobson.

*Medical Faculty of Harrisburg*—Drs. Riley, Henderson, Miller, Wiestling and Roberts.

